# Improving Exposure Assessment Using Non-Targeted and Suspect Screening: The ISO/IEC 17025: 2017 Quality Standard as a Guideline

**DOI:** 10.3390/jox11010001

**Published:** 2021-01-26

**Authors:** Juliana Monteiro Bastos da Silva, Jade Chaker, Audrey Martail, Josino Costa Moreira, Arthur David, Barbara Le Bot

**Affiliations:** 1 Oswaldo Cruz Foundation (FIOCRUZ), National School of Public Health, Leopoldo Bulhões, 1480, CESTEH, Rio de Janeiro 21041-210, Brazil; julianabastosdasilva@ensp.fiocruz.br (J.M.B.d.S.); josino.moreira@fiocruz.br (J.C.M.); 2 Univ Rennes, Inserm, EHESP, Irset (Institut de Recherche en santé, Environnement et Travail)—UMR_S 1085, F-35000 Rennes, France; jade.chaker@ehesp.fr (J.C.); audrey.martail@ehesp.fr (A.M.); barbara.lebot@ehesp.fr (B.L.B.)

**Keywords:** non-targeted, suspect screening, quality management, environmental health, ISO/IEC 17025, high-resolution mass spectrometry, quality standard

## Abstract

The recent advances of novel methodologies such as non-targeted and suspect screening based on high-resolution mass spectrometry (HRMS) have paved the way to a new paradigm for exposure assessment. These methodologies allow to profile simultaneously thousands of small unknown molecules present in environmental and biological samples, and therefore hold great promises in order to identify more efficiently hazardous contaminants potentially associated with increased risks of developing adverse health outcomes. In order to further explore the potential of these methodologies and push the transition from research applications towards regulatory purposes, robust harmonized quality standards have to be implemented. Here, we discuss the feasibility of using ISO/IEC 17025: 2017 as a guideline to implement non-targeted and suspect screening methodologies in laboratories, whether it is for accreditation purposes or not. More specifically, we identified and then discussed how specificities of non-targeted HRMS methodology can be accounted for in order to comply with the specific items of ISO/IEC 17025: 2017. We also discussed other specificities of HRMS methodologies (e.g., need for digital storage capacity) that are so far not included in the ISO/IEC 17025 requirements but should be considered. This works aims to fuel and expand the discussion in order to subsidize new opportunities of harmonization for non-targeted and suspect screening.

## 1. Introduction

Exposure assessment is the process of measuring or estimating the intensity, frequency, and duration of human exposures to an environmental agent [1]. Exposure assessment to environmental organic contaminants such as pesticides, pharmaceuticals or personal care products (e.g., UV filters, biocides) can be performed directly in human (i.e., collection of biological matrices such as blood, urine) or in environmental matrices in order to identify contaminant sources, transformation, transport and release mechanisms. So far, exposure assessments of organic contaminants have mainly relied on targeted methods where only a limited number of selected substances are analyzed. However, the diversity of chemicals we are facing (up to hundreds of thousands are currently in use in the human population [2]) requires to develop new methodologies to improve our capability to identify more efficiently hazardous contaminants associated with increased risks of developing adverse health outcomes.

In the last few years, the advances of new technologies based on high-resolution mass spectrometry (HRMS), usually coupled to liquid or gas chromatography, and omics-based methodologies have been implemented to profile thousands of unknown small molecules (mass-to-charge ratio comprised between 50 and 1500) in complex samples [3,4]. Some of these small molecules profiled with HRMS include organic contaminants mixtures, and all their transformation products present in the environment and that can accumulate in humans through different routes of exposure. Hence, these new approaches based on non-targeted analyses (NTA) and suspect screening (SS) strategies hold great promises to help identifying emerging substances or characterizing mixtures in samples without requiring a predefined list of compounds of interest [2]. More specifically, the SS strategy consists of looking for suspects which are known compounds (“known unknowns”) in terms of chemical name and structure which are expected to be present in a sample [5]. The list of suspects can be in theory unlimited but in reality, it usually arises from a systematic prioritization strategy. NTA aims to detect “unknown unknown” chemicals without any initial hypothesis to identify potential new markers of exposure [3]. However, it must be noted that it is hardly possible to avoid all initial hypothesis, since choice of sample preparation and analysis method enforce restrictions on the observable chemical space (e.g., eliminating compounds when using selective sample preparation methods or seeing mainly volatile compounds when using gas chromatography); therefore, NTA truly aims to limit restrictions as much as possible.

**Figure 1:**
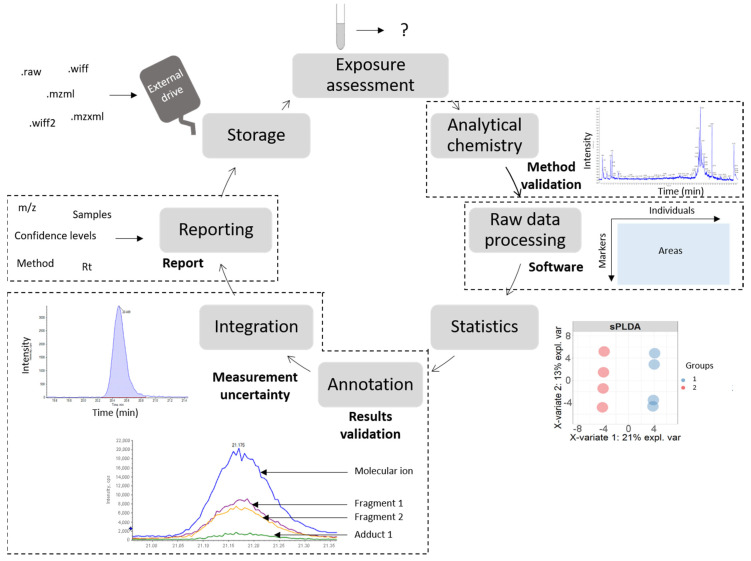
Steps of the non-targeted workflow requiring harmonization in order to be adapted for the ISO/IEC 17025:2017 quality standard (circled in dashed black lines). These steps are further discussed in the paragraphs indicated circled in orange solid lines.

In order to apply NTA and SS methodologies in routine analyses or for regulatory purposes, specific requirements (i.e., quality standards) have to be implemented to ensure the reproducibility and robustness of the results produced [6]. To this end, some international and European initiatives have started to encourage harmonization and validation of common tools and methods, such as the Network of reference laboratories, research centers and related organizations for monitoring of emerging environmental substances (NORMAN) and the Human Biomonitoring for Europe initiative (HBM4EU) [3]. As policies are mostly elaborated using evidence-based approaches, there is an additional need to harmonize practices amongst laboratories in order to allow comparison of results. Importantly, these harmonization practices should not be directed to implement standardized analytical techniques or data treatment processing between laboratories (mainly because innovation is still acutely needed in this emerging field) but rather should be oriented to the implementation of harmonized quality assurance (QA) procedures to evaluate the performance of the methods and the reporting of results. The need for QA procedure regards all the steps of NTA/SS workflow, as presented in Figure 1.

This harmonization task is extremely challenging considering the need to optimize and adapt all the steps required to perform NTA depending on the developed method, including the generation of data, the use of bioinformatics tools such as MZmine2 [7] or XCMS [8] to treat complex HRMS raw datasets or the annotation process [9]. There are some tools available to perform this workflow up to pre-annotations (with manual curation still required), such as Compound Discoverer [10], XCMS [8], or MS-DIAL [11]. These tools must still be optimized by choosing the appropriate steps and parameter values depending on the application (e.g., choosing to add or remove blank subtraction or area normalization, setting the noise level depending on the matrix complexity and potential contamination levels, etc.). Among all steps, the annotation/identification process (i.e., providing a putative/confirmed identity to a signal present in HRMS datasets) is the current bottleneck of SS and NTA. There is at present no tool available to fully automatize this step (i.e., manual curation still required), which means that a large portion of HRMS is returned partially annotated. This annotation process is complex and tedious, and requires providing solid information such as isotopic pattern, retention time and MS/MS fragmentation pattern in order to provide confidence in the annotation. As a final step and when possible, injection of reference standard compounds allows unambiguous identification of features (a feature is defined as retention time x *m*/*z* associated with an abundance (area/intensity)) [12]. This complex workflow relying on different expertise (analytical chemistry, bio-informatics) contributes to the lack of harmonized guidelines for QA in the emerging field of NTA/SS when compared to what is available for targeted analyses.

Despite the required initial investment (both human and financial resources), implementing QA management approaches can improve the robustness of results and discoveries for a research laboratory. Hence, this strategy can be considered as payback on the long-term investment. Within the types of matrices that an environmental health laboratory may handle (water, air, dust, and biological matrices), the regulation and demands regarding water analyses are the most evolved. For instance, in many European countries, laboratories that develop sanitary control activities have to demonstrate the existence of a QA system through an accreditation seal.

To ensure that quality standards are applied, accreditation and continuous improvement have become an intrinsic part of the discourse of many laboratories. An accreditation is a third-party attestation related to a conformity assessment body conveying formal demonstration of its competence to carry out specific conformity assessment tasks [13]. A laboratory can be accredited in many frameworks, with ISO/IEC 17025 being one of the most common ones. ISO standards have been established as continuative models for quality systems and as so, the changes in the environment, technology, and legislation worldwide make ISO standards periodical reviews necessary [14]. In 2017, a third version of ISO/IEC 17025 was published. This article will be based on this latest edition to perform its analysis.

The ISO/IEC 17025 establishes the criteria for those laboratories that wish to demonstrate their technical competence, that have an effective quality system and that are able to produce technically valid results, promoting confidence in their work both nationally and around the world to attest the competence of laboratories to perform tests and/or calibrations, including sampling [15].

The goal of this article is to discuss points of ISO/IEC 17025 that specifically differ for NTA/SS research compared to targeted methods, since they are the applications for which this framework was built. These points are all contained within Section 7 (Process Requirements) of the ISO/IEC 17025 Standard Structure. Therefore, other sections will not be further discussed (see Table 1).

Items from Section 7 of the ISO/IEC 17025 will be used to consider the feasibility of using this standard as a guideline to create harmonized QA/QC procedures to implement NTA/SS methodologies in laboratories, whether it is for accreditation purposes or not. Since this quality framework’s applications are broad, this work aims to identify and discuss how specificities of NTA and SS methodologies can be accounted for to comply with the specific items of ISO/IEC 17025: 2017 in the broad context of exposure assessment. We also discussed other needs of HRMS methodologies that are not explicitly included in the ISO/IEC 17025 but should be considered.

## 2. Materials and Methods

In order to check the compatibility of the ISO/IEC 17025 with the specificities of NTA methodologies applied to organic contaminants, a detail study of the guideline was developed, analyzing each ISO requirement. Although this analysis was guided towards assessing exposure to organic compounds, similar reasoning can be applied for inorganic chemicals such as are rare earth elements, metals, etc. From this step, a scale was established defining each item as “Compatible”, “Adaptable” or “Not applicable”. After the definition of the scale, we have found that, with adaptations, every item of ISO/IEC 17025 framework was applicable to NTA/SS studies and we have therefore eliminated the “Not applicable” level.

Each requirement identified as “adaptable” was classified in five “discussion topics”: measurement uncertainties, validity of the method, validity of the results, report, software and an adaptation strategy to comply with the framework’s requirement was proposed for each of them. Items defined as “Compatible” were not discussed further as they are not specific to NTA/SS studies and do not require any specific adaptations compared to what is done for targeted approaches. The discussion topic about “Measurement uncertainties” appears in items 7.2.1 and 7.6, but is only discussed in Section 7.2.1 (see Table 2).

It is important to highlight that several guides have been elaborated to help the implementation of ISO/IEC 17025 in laboratories. These documents are published by the International Accreditation Forum (IAF) members and signatories for application in each country and can be mandatory to ISO/IEC 17025 accreditation. For this analysis, when clarification of the requirements was needed, some guidelines published by this accreditation bodies were also taken into consideration, such as LAB GTA 86 of the Comité Français d’Accréditation (COFRAC) [16] and LAB 12 of the United Kingdom Accreditation Service (UKAS) [17]. Besides ISO/IEC 17025, additional requirements may be placed on laboratories, depending on their specific activities, to ensure that quality data are being reported. An example would be the 2002/657/EC European Union Decision [18] on laboratories involved in veterinary drug residue analyses. Although these norms may set performance criteria for analytical methods, for instance, they are not part of the scope of this study, which focuses on requirements initially found in ISO/IEC 17025.

## 3. Results

### 3.1. Measurement Uncertainties

- “Measurement uncertainties” in “7.2 Selection and verification of methods”

The normative requirements of ISO/IEC 17025 regarding measurement uncertainties are focused on quantitative results, which is coherent in the case of targeted analyses, as these results are usually the main pursued objective. However, NTA techniques lead to the production of results that rely on both qualitative (e.g., annotation of unknown features in HRMS datasets) and quantitative (e.g., inter-individual relative comparison) aspects. To fulfill the normative requirements of ISO/IEC 17025, we consider that measurement uncertainty must include both the qualitative and quantitative aspects.

The qualitative aspect (i.e., the annotation process of HRMS datasets) is currently one of the bottlenecks of NTA. It is usually considered than less than 10% of HRMS dataset can be annotated [19]. Identification relies mainly on structural information such as MS/MS fragmentation pattern but also on other criteria such as the *m*/*z* accuracy, retention time or isotopic pattern. Confidence levels from 1 to 5 based on information generated during the analytical process (*m*/*z*, retention time, isotopic pattern, MS/MS data) have already been proposed for xenobiotic annotation by Schymanski et al. [6]. Hence, the reporting of qualitative criteria should include all the relevant information and provide a confidence level based on these recommendations or other initiatives (e.g., Metabolomics Standard Initiative [20,21,22].

It is also important to mention that in order to help the annotation process, the strategy to find relevant information to annotate unknown features can be adapted to the research topics, the matrix, and of course the analytical technique. Specific databases exist for environmental applications (e.g., MassBank [23]) and human applications (e.g., HMDB [24], Drugbank [25], Exposome explorer [26]) depending on the matrices such as Serum Metabolome [27] or Urine Metabolome [28] for human serum or urine in the case of biological matrices, or FOR-IDENT [29] for water in the case of environmental matrices.

Quantitative criteria are also challenging to define for NTA/SS. These methodologies only provide semi-quantitative data (i.e., comparison of fold changes of normalized area between individuals) as opposed to targeted analyses, which can provide quantitative data using internal standards to correct analytical variabilities. So far, quantitative criteria for NTA/SS mainly rely on area/intensity integration. Uncertainty of area quantification can be estimated using different normalization strategies based on batch correction using quality controls (i.e., composite samples) reinjected periodically during and between batches, by normalizing to the total area or using similar strategies to targeted analysis with a set of molecules considered representative. Recently many strategies have been proposed and used for the (semi-) quantification to obtain a concentration estimation across compounds and samples [30]. In any case, the strategy used to provide any quantitative data should be well reported (e.g., type of normalization used, number of QCs injected, identity and concentrations of internal standards). It must also be noted that area integration is also conditioned by the chosen peak picking software and parameters used, which should also be reported.

- “Measurement uncertainties” in “7.6 Evaluation of measurement uncertainty”

Evaluating measurement of uncertainty must also include a step of identification of influence factors. Indeed, “LAB GTA 86—Recommandations pour la mise en œuvre de la norme NF EN ISO/IEC 17025 en vue de l’accréditation des Laboratoires”, published by COFRAC and “LAB 12-The Expression of Uncertainty in Testing” published by UKAS both clarify that when the framework is not explicit about the uncertainty assessment approach, and there are no additional recommendations in the guides, a simple judgment based on the identification of the factors likely to influence the result of measurement is acceptable.

To accomplish this identification, an Ishikawa diagram would be an appropriate way to comply with the requirement. This diagram represents the measurement process and the elements that influence the outcome. According to LAB GTA 86, if certain factors have an influence considered insignificant, the laboratory should specify the elements that prove it.

**Figure 2:**
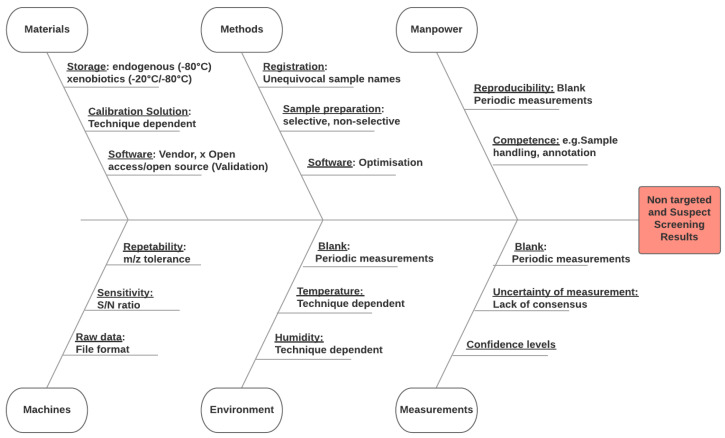
Non-exhaustive Ishikawa diagram for non-targeted and suspect screening methodologies.

Several factors are particularly or exclusively influent in the case of NTA/SS approaches to investigate exposure assessment. These influence factors are presented in Figure 2 and can be retained in six main categories:

Materials can refer to samples, reference or standard solutions, or software tools used for acquisition and/or data treatment. Storage of samples is a critical point as temperature must be controlled to avoid analyte loss or degradation. Since compound-specific freezing study would be a limited option for a NTA study, all precautions must be taken to protect the sample from degradation as a whole. A storing temperature of −80 °C is usually preferred and recommended, particularly for biological matrices. Calibration solutions are also a point to consider carefully, as NTA/SS methods using HRMS rely on accurate mass. Frequency of calibration can vary based on the used instrument: time-of-flight (TOF) instruments usually offer real-time calibration (up to a calibration for each sample), whereas Orbitrap instruments may be calibrated weekly. Another example worth discussion is the Laboratory information management system (LIMS), which has to be dimensioned for NTA/SS data sizes, as these types of studies tend to generate large amount of information compared to targeted studies.

Machines have several inherent characteristics that affect the outputted results. For example, repeatability is especially critical in NTA/SS approaches, as loss of analytical sensitivity and drift in retention time can hinder efficient annotation or impair inter-individual comparisons. Different HRMS techniques offer varying levels of repeatability regarding mass accuracy, as mass tolerance is usually 10–15 ppm on TOF and 5 ppm on Orbitrap [5]. When performing exposure assessments, low-abundant signals can become harder to distinguish from the background noise in the course of the batch. This ties with issues of sensitivity, which is another critical instrument characteristic, and requires rigorous schedules and procedures for cleaning to avoid response decay. Lastly, data produced by instruments will have a particular format (RAW, wiff, etc.). This format can be a constraint when using certain data processing software tools, as each tool accepts a limited number of input formats. Data can be converted to open formats such as mzML or mzXML, which are accepted by most open source NTA data processing tools. The conversion process should however be investigated to ensure that no data is lost or altered.

Methods and procedures should be evaluated with the specifics of NTA/SS in mind regarding compound coverage, selectivity, and sensitivity. Since these approaches aim at creating an analytical fingerprint of samples, it should be possible to investigate a posteriori exposure assessments months or years after the analysis took place. Samples should therefore be registered with an unequivocal name that allows tracing back to its characteristics (origin, date, operator, etc.) as well as analytical techniques used for the analysis. Regarding these techniques, recommendations have already been made for NTA research for quality assurance/quality control, which heavily depend on the type of method used [31,32]. Indeed, choosing liquid or gas chromatography, ionization polarity, chromatographic conditions, sample preparation technique, and data processing software are critical points when using NTA since it can widely influence the results generated [33]. Methodological aspects should be thoroughly investigated to characterize their advantages and limitations, which is critical for result interpretation. To this end, reference materials can be used to determine a method’s observable chemical space, such as what was performed for EPA’s Non-Targeted Analysis Collaborative Trial (ENTACT) using the National Institute of Standards and Technology (NIST)’s Standard Reference Material (SRM) 2585 and human serum (NIST SRM 1957) [34]. The data processing software is also a critical point when using NTA since it influences the returned data. Such tools should be thoroughly investigated to determine the optimal parameters for each intended application. Similarly, tools and libraries used in the identification process will affect the obtained annotations, and should therefore be investigated, or at least reported.

Environment encompasses all that surrounds the analysis process and is less specific to NTA/SS, as regular blank measurements [5], temperature [35] and humidity controls are always recommended.

Manpower underlines the need for operators to be trained to handle samples intended for NTA and the resulting data. For instance, quality control procedures such as repeated blank or reference materials measurements are needed to ensure reproducibility. Moreover, annotation is a step specific to NTA/SS that requires proper training and is time-consuming. Avoiding cross contamination is also a key aspect on which analysts should be trained, since external sample contamination is generally harder to identify in NTA/SS studies.

Measurements in NTA are not as well defined as in targeted analyses. Indeed, there is, to date, a lack of consensus regarding measurement uncertainty in the NTA community. As specified earlier, qualitative and quantitative criteria can be used to evaluate NTA measurement uncertainty, but no guidelines exist yet to frame this evaluation specifically in the context of exposure assessment. However, confidence levels have been constructed to qualify annotations [6]. These levels range from 5 (lowest confidence) to 1 (highest confidence, annotation confirmed with a standard) offer a homogenized and efficient way to communicate the confidence that can be put in any given annotation.

Points that could influence the results determined by the laboratory (within these six categories) must be identified clearly on such a diagram or any other similar tool, in order to comply with ISO/IEC 17025’s requirement regarding evaluation of measurement uncertainty. Additionally, the points discussed here are those that are specific to NTA/SS approaches, but must be completed with other usually considered measurement uncertainty sources, such as materials and mobile phase purity and grade, injection volume, flow rate, or column choice.

### 3.2. Validity of the Method

Although method validation is well defined for targeted approaches, it is not the case for NTA/SS because these methodologies deal with a tremendous amount of unknown features (up to several thousand within one dataset) as opposed to a limited number of chemicals for multi-residue methods. It is often recommended for NTA to use minimal sample preparation to avoid the loss of compounds of interest. However, using more selective sample preparation is often necessary for exposure assessment purposes to decrease analytical interferences and detect very low-abundant xenobiotics in complex matrices, in particular with UHPLC-ESI-HRMS platforms which suffer from ion suppression [36]. Hence, method validation for NTA methods is a critical point to be discussed, as it is intrinsically different from targeted methods. This consideration is added to the usual concerns of sample preparation, as compatibility with the research question and the matrix.

To answer the normative requirements on method validation, one possibility could be to include targeted strategies to validate the sample preparation using a set of representative molecules in order to provide recovery, method detection and quantification limits, repeatability, etc. to assess NTA method performances [36]. The set of compounds has to be carefully chosen and should include chemicals with a wide range of physical-chemical properties [36]. Besides quantitative criteria, qualitative criteria have to be provided to validate the sample preparation. For instance, the presence or absence of features (or their fold changes) across different sample preparation methods as well as makers found in preparation blanks can be assessed using statistical comparisons and should be reported. Ideally, the identity of features specific to a sample preparation method should be reported to better characterize its coverage. Statistical tools such as principal components analysis (PCA) or *t*-tests can be used to detect abnormalities or drifts within batches, and potentially identify some causes [35]. This is especially the case when a composite quality control sample is used, since its signal should be stable.

Another specificity of NTA methods is software optimization. Indeed, establishing the list of features for each sample using raw data processing software is a critical step to avoid false negatives. To answer the standard’s requirements regarding software validation, a list of application-specific criteria (technical, financial, etc.) should be defined to choose and then evaluate possible data treatment solutions. The technical criteria can be based on a list of molecules as for targeted methods, and can include ease of implementation, data processing time, false negative rate, etc. To attain these objectives, as discussed earlier, it is possible to build the raw data processing workflow (peak picking and deconvolution algorithms, normalization, blank subtraction, etc.) and optimize the related parameters (noise threshold, *m*/*z* and retention time tolerance, etc.). Similarly, the identification process is specific to these approaches, and can be performed by a software tool that should be optimized for the intended application. During this process, a list of parameters will be compared between features found in the sample and compounds issued from a database. This list of parameter includes *m*/*z*, retention time (in the case of SS), isotopic pattern or ratio, fragmentation pattern or ratio, etc. Therefore, some parameters must be given such as value of *m*/*z* accuracy, retention time tolerance (in the case of SS), etc. The complexity of the raw data processing and identification steps and their specificity to these new approaches justify the need for a particular focus on them. Indeed, a lack of software optimization to perform these steps can lead to high false negative rates due to poor peak picking and/or partial identification [9]. Further software normative requirements are discussed later in the text.

According to ISO/IEC 17025, the techniques used for method validation can be one or a combination of several options, including using reference standards or reference materials (RM), which should be traceable to national/international standards as per the norm’s requirement. According to GEN REF 10, in order to be traceable, the reference materials should be supplied by a national metrology laboratory under the cover of the mutual recognition agreement of the international committee for weights and measures.

ISO/IEC 17025 requires that laboratories shall reconsider systematically and periodically the relevance of its method of performance monitoring. As the NTA community is growing and evolving, tools such as collaborative trials proposed by the NORMAN network could become the tools to answer this requirement. These trials have a different aim than the targeted proficiency testing, as the goal is for each participating laboratory to contribute, with its methods, instruments and expertise, to the knowledge regarding exposure of a given matrix. NTA collaborative trials are currently available through different organizations, such as the Norman Network, or EPA’s Non-Targeted Analysis Collaborative Trial [34,37].

### 3.3. Validation of Results

In order to ensure the quality and validity of results, systematic monitoring has to be performed. Regarding this topic, ISO/IEC 17025 gives a non-exhaustive list of steps to implement, most of them compatible with NTA methods. In this last case, three particular parameters can be used.

Firstly, blank measurements have to be performed regularly, as they are necessary to avoid false positives. Secondly, to monitor performance and check measurement stability, reference materials, such as internal standards, should be used to ensure the validity of NTA results. Thirdly, the injection of composite quality controls (mixing a small quantity of every sample) at regular intervals gives an information on measurement stability at the NTA scale [38]. Appropriate frequency for these measurements should be defined based on the instrumentation robustness. Blank measurements must be performed with each batch and a set of internal standards (deemed representative of the observable mass and physical-chemical properties ranges) may be systematically used (in each sample) to ensure the proper conduct of sample preparation and to check for any analytical drift during the batch analyze. Injection of composite quality controls can be performed before the batch injection to equilibrate the instrument and every few samples (5–7) to compromise between total batch run time and thorough tracking of batch analysis [33,35].

### 3.4. Report

Reporting as required by ISO/IEC 17025 is possible for NTA. However, the NTA community has not yet come to a consensus regarding what is relevant to report, or its format, even though the results related to the annotation of the suspect is the most critical aspect. Hence, it should be mandatory to clearly report all the analytical parameters related to annotation process and provide a confidence level. As large amounts of information are generated, the independent parties should agree on a number of specific items that should appear in the report. Its format should be understandable and all reported information should be agreed with the customer to ensure that data necessary for the results’ interpretation is available. Moreover, the contextual information (e.g., sampling, storage conditions, sample preparation method, etc.) should be recorded. Additionally, if the laboratory intends to be accredited, any analytical step that is externalized must be in conformity with the requirements established by the laboratory. In addition, all issued reports shall be retained as technical records. This is further discussed in the next topic.

### 3.5. Software

Regarding software requirements, ISO/IEC 17025 states that information management systems should be validated for functionality, including the proper functioning of interfaces within the laboratory information management systems. The guideline also considers that all used software and libraries should be validated using a set of criteria determined by the user to ensure that all used software accurately perform the intended tasks. Off-the-shelf software in general use within its designed application range can be considered to be sufficiently validated. In NTA methodologies, there are many “ready-to-use” and open source software available but optimization must be performed. Furthermore, even though there is no universal global software tool to perform all data treatment required for NTA/SS, i.e., from the raw data processing to the finalized identification, it is worth mentioning that very promising workflows are emerging to harmonize the whole process, e.g., Workflow4Metabolomics [39].

In NTA screening, chromatograms are screened for features using suitable peak finding software. To validate these results, ISO/IEC 17025 demands several verifications, e.g., software’s lock, to guarantee the correspondence between mass and element. Other validation criteria for the software, such as tolerance for false negatives, integration repeatability, or speed of implementation, are up to the user to determine, as there are no further recommendations from the framework. Furthermore, according to LAB REF 02—Exigences pour l’accréditation des laboratoires selon la norme NF EN ISO/IEC 17025 [40], the laboratory must ensure the accuracy of any external data that it uses, including databases, when they are used to develop the laboratory’s activities.

### 3.6. Non-Targeted Critical Challenges

As observed, and expected, the ISO/IEC 17025 requirements are broad, but they do not treat directly specificities that are critical for NTA methodologies. These specificities are mostly linked to the innovation factor inherent to the all-new analytical methods. These new concepts are as important as interesting and should be raised to start and fuel a discussion regarding NTA/SS QA management needs.

In order to progress on the path of quality assurance of NTA methods, quality actors should be aware of these challenges. After the analysis realized in this paper, some points have been identified as critical in the drafting of new regulations in the area.

#### 3.6.1. Digital Archives

HRMS-based methods generate “full scan” fingerprints which offer the possibility of “coming back in time” many years later to check a posteriori the potential presence in these fingerprints of a specific environmental contaminants suspected to be associated with a studied health outcome. Hence, appropriate data storage is critical for NTA methodologies.

To fulfill this purpose, it is necessary to store a Chemical Archive within a data repository to complement the storage of biological samples. Such repositories also allow data exchange within the scientific community, leading to increased efficiency, robustness, and confidence in findings. They also allow the storage of metadata (sample preparation, analytical methods, processing software, etc.), which is critical for result interpretation. The NORMAN network’s sample freezing platform, for instance, allowed the screening of 670 antibiotics and 777 REACH chemicals in various sample types from the Black Sea, including biota and seawater samples [31]. Many other repositories are available, such as MetaboLights [41], MetabolomeXchange [42], or MassIVE [43].

Although there is a chapter in the quality standard regarding the control of data and information management, these crucial issues are not mentioned and would be important to consider at the time of the quality standard implementation or even at the time of an audit.

#### 3.6.2. Sample Identification and Registration

As NTA methods aim at a close-to-exhaustive characterization of a sample, the review of acquired data should be possible several months or years later, as discussed previously. Samples should therefore be registered with an unequivocal name that allows tracing back to its characteristics (origin, date, operator, etc.) as well as analytical techniques used for the analysis. The use of a unique laboratory number that is continuously used in all files names and documents would be useful [5]. Additionally, information regarding sample preparation and sample analysis should be conserved and easily traceable using the unique sample identifier, as well as sampling conditions, sample history and freeze/thaw cycles, if available.

#### 3.6.3. File Conversion to Open Formats

Since users have little power over the duration of vendor software support, vendor formats may become obsolete at any given time. To avoid issues of unreadable formats, and therefore loss of data, data produced in NTA/SS methodologies must be made available in open formats. All data generated should therefore be duplicated in an open format, using readily available software such as ProteoWizard’s MSConvert [44]. Reproducibility and readability throughout the entire archiving period must be assured.

#### 3.6.4. LIMS Compatibility with Non-Targeted Data

Another critical point of implementing quality standards in non-targeted methods is the compatibility with the LIMS, as it is a crucial part of the quality process. Due to the large volume of data generated, the LIMS should be able to cover all the needs related to the non-targeted needs. As LIMS are not typically engineered to handle such large amount of data, they can be used as a traceability tool to be linked with better-suited data repositories, which could answer the need for digital archives discussed earlier.

## 4. Discussion

The path to a broadened use of NTA/SS methods’ application for routine or official control purposes has to go through quality assurance tools. The application of quality guidelines would help the monitoring, controlling and improving reliability of NTA/SS findings the same way it did for the validation of targeted methods [35,45].

An experience of Dirnagl and colleagues [46] shows, from an ISO 9001 implementation experience in a research laboratory, that a structured approach to Quality Management has enormous potential to improve the quality of research without stifling creativity if done properly. However, critical points for its implementation should be considered, such as the terminology being alien to many scientists, and the introduction of a normative regulatory system applying additional pressure on scientists who already work in a competitive system. According to Buchta et al. [47], quality systems according to ISO 9001 and ISO 15189 give a solid basis for higher analytical laboratory performance.

Although these publications bring interesting points to discuss, it is important to highlight that ISO 9001 is not a laboratory specified standard, so it is understandable that its overall design did not match with the idiosyncrasies of research process.

In a paper published by Zapata-Garcia and colleagues in a university testing laboratory, the results of the ISO/IEC 17025 accreditation was positive. According to the group, this accreditation impacted on the rest of the research group’s activities by better control of equipment and the introduction of some changes in the way of working. These changes ensured the quality of the produced results, improving the research activities [48].

In the field of research, there are some initiatives of developing more suitable quality management systems tailored to academic needs [46]. Through both the research and service fields, NTA methods face an improvement opportunity by the application of quality standards. Some initiatives have been launched as the guideline “Use of non-targeted screening by means of LC-ESI-HRMS in water analysis” [5] in order to developing the method and interpreting the results. However, while it is a useful essay of harmonizing some practices, it does not meet the formalization level of normative standards.

## 5. Conclusions

In the context of an accredited laboratory already relying on this framework for targeted analyses, or of a team or a scientist’s individual will, the ISO/IEC 17025 framework can be a useful tool to formalize quality assurance and quality control procedures in NTA/SS studies. Although there are no items in the framework that are incompatible with these new methodologies, some adaptations must be made regarding the evaluation of method and result validity, measurement uncertainties, and processing software. Moreover, several critical aspects of NTA/SS approaches are not covered by this framework, such as the need for well-maintained chemical archives. Through the application of an adapted version of an already available quality management standard, regardless of individual methodological choices, results of exposure assessment research using NTA/SS approaches will gain in robustness, replicability, and transparency.

The harmonization of QA and QC procedures in NTA/SS approaches is directed to organic chemicals but similar procedures can be applied to inorganics analysis. The harmonization of QA and QC procedures in NTA/SS approaches for exposure assessment will allow robustness and confidence in the results of such studies regardless of the differences in analytical methodologies (sample preparation, analytical technique, etc.). These technological/analytical methodologies should not be standardized since their diversity ensures a wider coverage of environmental contaminants. As the harmonization of QA and QC procedures is a broad task that cannot be done at the scale of one laboratory, it is of critical importance to continue this discussion at a larger scale. This article is a reflection of the authors’ point of view that does not implies an auditor analysis. All analysis made took into account the experience and sound judgment of the researcher.

## Figures and Tables

**Table 1:** ISO/IEC 17025 Standard Structure. Points that apply to specific technical aspects of non-targeted and suspect screening approaches that are discussed in this work are in Section 7—Process requirements, Sections 7.2, 7.6, 7.7, 7.8, and 7.11, indicated in bold.

ISO/IEC 17025: 2017
1 Scope
2 Normative references
3 Terms and definitions
4 General requirements
5 Structural requirements
6 Resource requirements
7 Process requirements 7.1—Review of requests, tenders and contracts **7.2**—**Selection, verification and validation of methods** 7.3—Sampling 7.4—Handling of test or calibration items 7.5—Technical records **7.6**—**Evaluation of measurement uncertainty** **7.7**—**Ensuring the validity of results** **7.8**—**Reporting of results** 7.9—Complaints 7.10—Nonconforming work **7.11**—**Control of data and information management**
8 Management system requirements
Annex A Metrological traceability Annex B Management system options

**Table 2:** Classification of ISO/IEC 17025 adaptable requirements for non-targeted/suspect screening methodologies.

ISO Requirements				Discussion Topic	Non-Targeted and Suspect Screening Specificities
7. Process Requirements					
**7.2**	**Selection, verification and validation of methods**	7.2.1	Selection and verification of methods	**“Measurement uncertainties”**	Qualitative criteria: Annotation - *m*/*z* error (ppm) - Isotope pattern - Retention time (RT) - MS/MS
					Quantitative criteria - Inter-individual signal variation - Fold change - Concentration ranges
		7.2.2	Validity of methods	**“Validity of the method”**	Identification of influence factors - Sample registration - Sample preparation - Software optimization
					Method Validation Strategy - Implementation of targeted strategies using a set of representative compounds for recoveries and detection/quantification limits
					Use of traceable mass calibrant for HRMS platforms - Metrological traceability
					Participation in collaborative trials - Goal of collaborative trials - Participation opportunities
7.6	Evaluation of measurement uncertainty			**“Measurement uncertainties”**	Identification of influence factors - Ishikawa diagram (see Figure 2) - Identification confidence level 4
7.7	Ensuring the validity of results			**“Validity of results”**	Systematic monitoring of results - Blank measurements - Internal standards (ISTD) - Composite sample quality controls (QCs)
7.8	Reporting of results			**“Report”**	Definition of relevant information and format - Undefined amount of information to report - Lack of suited information systems
7.11	Control of data and information management			**“Software”**	Pre-processing and annotation software validation - Validation criteria

## Data Availability

Not applicable.
